# Principles of Mutual Information Maximization and Energy Minimization Affect the Activation Patterns of Large Scale Networks in the Brain

**DOI:** 10.3389/fncom.2019.00086

**Published:** 2020-01-09

**Authors:** Kosuke Takagi

**Affiliations:** Independent Researcher, Saitama, Japan

**Keywords:** functional connectome, information processing, mutual information, network energy, activation pattern, large scale brain network

## Abstract

Successive patterns of activation and deactivation in local areas of the brain indicate the mechanisms of information processing in the brain. It is possible that this process can be optimized by principles, such as the maximization of mutual information and the minimization of energy consumption. In the present paper, I showed evidence for this argument by demonstrating the correlation among mutual information, the energy of the activation, and the activation patterns. Modeling the information processing based on the functional connectome datasets of the human brain, I simulated information transfer in this network structure. Evaluating the statistical quantities of the different network states, I clarified the correlation between them. First, I showed that mutual information and network energy have a close relationship, and that the values are maximized and minimized around a same network state. This implies that there is an optimal network state in the brain that is organized according to the principles regarding mutual information and energy. On the other hand, the evaluation of the network structure revealed that the characteristic network structure known as the criticality also emerges around this state. These results imply that the characteristic features of the functional network are also affected strongly by these principles. To assess the functional aspects of this state, I investigated the output activation patterns in response to random input stimuli. Measuring the redundancy of the responses in terms of the number of overlapping activation patterns, the results indicate that there is a negative correlation between mutual information and the redundancy in the patterns, suggesting that there is a trade-off between communication efficiency and robustness due to redundancy, and the principles of mutual information and network energy are important to network formation and its function in the human brain.

## 1. Introduction

Interactions of ~100 billion neurons, which are a part of the human brain, maintain its functions within a hierarchical and modular network structure (Azevedo et al., 2009; Meunier et al., 2010; Park and Friston, 2013). Empirical evidence demonstrate that a stimulus for local excitatory neurons at a cellular level can be etiologically associated with large-scale brain activity, which may propagate through numerous neuronal interconnections (Beggs and Plenz, 2003; Beggs, 2008; Lee et al., 2010; Fenno et al., 2011; Tagliazucchi et al., 2012). Over the years, studying task evoked brain activity via whole-brain imaging has been successful in mapping specific cognitive functions onto distinct regions of the human brain (e.g., Kanwisher et al., 1997).

Furthermore, several studies that have examined the brain's responses to more complex tasks, reported that various cognitive functions arise from interactions between regions of the brain rather than independent single activities in distinct regions of the brain (Ghazanfar and Schroeder, 2006; Bressler and Menon, 2010). In the large-scale networks of the human brain, activation signals from segregated and specialized regions are integrated in information processing (Tononi et al., 1994; Hilgetag and Grant, 2000; Sporns, 2013). Thus, the brain can be conceptualized as an information processing system, hereby successive patterns of activation and deactivation in multiple distributed regions constitute integrated information processing. Furthermore, the brain must adapt to changing environments, so these processes might be optimized to ensure rapid and flexible response (Bassett et al., 2006; Kitzbichler et al., 2009; Clark, 2013; Park and Friston, 2013; Mnih et al., 2015). On the other hand, the brain is limited by its energy requirements and by other biological realities (Bullmore and Sporns, 2012). Thus, the need to maximize efficiency of information processing and minimize total energy consumption may regulate the mechanisms underlying the structure and the function of the brain (Linsker, 1990; Friston, 2010; Bullmore and Sporns, 2012).

This argument is known as the energy efficiency hypothesis, which covers a wide range of activities from the cellular level of neurons to the global level observed at the scale of the whole brain (Bullmore and Sporns, 2012; Yu and Yu, 2017). Evidence for this hypothesis has shown that the energy constraints and limitations may affect multiple aspects of the brain neurons by inducing efficient activities (e.g., Niven and Laughlin, 2008; Tomasi et al., 2013; Yu and Yu, 2017). The energy consumption models of neurons have especially been studied in detail, and they have revealed the requirements from energy efficiency effects on neuronal activities or on those at the cortical level follow the energy efficient principle (Wang et al., 2008, 2015, 2018; Wang and Wang, 2014).

In the present paper, I present evidence that this pattern is especially the case in the information integrating processes in a large scale network, demonstrating that maximization and minimization principles guide the network structure and activation patterns of the human brain. Based on functional connectome data acquired using resting-state functional MRI (fMRI) (Sporns, 2002; Fox and Raichle, 2007; van den Heuvel et al., 2008; Greicius et al., 2009; Biswal et al., 2010; Van Dijk et al., 2010; Brown et al., 2012), I simulated information transfer by applying randomly activated signals to a network represented by brain connectivity matrices (Takagi, 2018). I measured mutual information (Linsker, 1990) between random stimulus signals and their responses and also quantified the network energy associated with these activities (Hopfield, 1984; Hinton and Salakhutdinov, 2006). By varying the functional connectivity network between noisy and sparse states, I showed an explicit correlation between these quantities. The results suggest that there is an optimal intermediate between these states, whereby mutual information is maximized and the network energy is minimized.

On the other hand, evaluation of the network structure around this optimal intermediate state revealed some features that are characteristic of the functional connectome, such as small-world and criticality (Watts and Strogatz, 1998; Achard et al., 2006; Bassett and Bullmore, 2006; Hagmann et al., 2008; van den Heuvel and Sporns, 2011; Takagi, 2017, 2018). These characteristic attributes are thought to explain the brain's rapid adaptive responses to external stimuli and the robustness of its internal communication (Kitzbichler et al., 2009; Chialvo, 2010; Tagliazucchi et al., 2012). Experiments at a cellular level demonstrated that neuronal firing successively propagated similar to neuronal avalanches; however, their size has no characteristic scale (Beggs and Plenz, 2003; Beggs, 2008). However, analyzing the fMRI dynamics revealed that the dynamic and statistical properties which regulate activation events on a scale of the whole brain were identical (Tagliazucchi et al., 2012). This feature of the dynamics appeared across multiscale from the cellular level to the brain macro scale is explained by the feature of the criticality, the absence of the characteristic scale (Beggs and Plenz, 2003; Beggs, 2008; Tagliazucchi et al., 2012). Additionally, optogenetic methods combined with fMRI facilitate direct visualization of the global level activity caused by local neuronal excitation (Lee et al., 2010; Fenno et al., 2011). Besides the absence of a characteristic scale for these dynamical activation events, an identical feature that is predicted from the criticality can be confirmed in the functional network structure, which was constructed using the spatio-temporal correlations between brain regions. To illustrate, network node degree statistics exhibit the distribution characteristic similar to the critical phenomenon (Achard et al., 2006; Bassett and Bullmore, 2006; Hagmann et al., 2008; Takagi, 2017). Moreover, within these networks, strongly connected pathways compose core structures with highly connected hub regions that modulate information processing in the brain (Hagmann et al., 2008; van den Heuvel and Sporns, 2011). Processing in these regions may control multiple brain functions (Rubinov and Sporns, 2011). The results show that, to ensure optimal efficiency and energy use, the network structure converges on this characteristic state exhibiting small-world and criticality.

Further analyses of the simulation results of the information transfer model revealed direct evidence that this characteristic state regulates activation patterns (Takagi, 2018). In the simulation, response patterns exhibited redundancy in that they contained repeatedly co-activated regions with different stimulation signals. In the cerebral cortex, activation patterns exhibit overlapping that can be measured as the proportion of regions activated equally in the different patterns. This can in turn be related to cognitive processes, such as memory retrieval (Haxby et al., 2001; Kumaran et al., 2016). While functionally overlapped regions may offer robustness in communication and facilitate adaptation (Whitacre, 2010; Bassett et al., 2018), excess overlapping causes interference. This can result in decoding difficulties that can be costly in terms of metabolic consumption (Kumaran et al., 2016). In the present study, the average of the overlapping numbers depended on the mutual information and the network energy. It showed the negative correlation to the mutual information and the correlation to the network energy. The results imply that the principles of mutual information and network energy strongly affect the activation patterns and the underlying structure of the functional network in the brain.

On the other hand, it is known that the functional connectivity is flexible within certain dynamics; for example, alterations in diseased brains, or the break-down from criticality in the unconsciousness, have been reported (Tagliazucchi et al., 2016; Song et al., 2019). As such, the robustness of the simulation results in this paper are validated, in comparison to different datasets, such as those with different sized matrices with different sets of nodes and those that were constructed from the structural connections based on diffusion tensor imaging (DTI) (Sporns et al., 2005; Brown et al., 2012). They also indicate that the relationships between the mutual information, the energy of the activation, and the activation patterns that emerge are stable within these networks as well.

## 2. Materials and Methods

### 2.1. Connectome Datasets and Information Transfer Model

#### 2.1.1. Functional Connectome Datasets

I modeled information transfer in the large scale network of the human brain using a functional connectivity matrix constructed from fMRI observation (Takagi, 2018). As explained in the introduction, a stimulus at the cellular level can trigger avalanche events at a whole-brain scale due to the characteristic features of the critical phenomenon. Whole-brain scale observation through fMRI revealed that neighboring voxels overlapping in their dynamics show similarities in time series data, because of successive appearances of these events (Calhoun et al., 2009; Smith et al., 2011; Smith, 2012; Tagliazucchi et al., 2012). Therefore, it is possible that the information relevant to the underlying brain activity is compressed (Tagliazucchi et al., 2012). Furthermore, a relevant network model is constructed by extracting nodes, through independent component analysis or clustering voxels on the basis of the similarity (Calhoun et al., 2009; Smith et al., 2011; Smith, 2012; Tagliazucchi et al., 2012).

To accurately analyze the network in the whole-brain scale, hundreds of nodes are typically utilized to construct a network from fMRI time series data (Smith, 2012; Finn et al., 2015). The validity of the network construction is then indicated by the robustness for different individual subjects (Smith, 2012; Finn et al., 2015). Here, the validation of the pre-processed network datasets was demonstrated by the results of my previous study using the same dataset, which reported a stable statistical significance regarding the network structure (Takagi, 2018). Additionally, the robustness of the current study and the consistency with other studies will be discussed in the final section.

For each combination of single regions in the brain, the connectivity matrix was described as a matrix (*w*_*ij*_), whereby (*i, j*) represented the connection weight between regions denoted as *i* or *j*. For the time series data of the fMRI image, the connectivity was calculated as the Pearson correlation coefficient between voxels corresponding to these regions. In the present study, I used the preprocessed connectivity matrices, which are available from http://umcd.humanconnectomeproject.org/: the website of the USC Multimodal Connectivity Database (Brown et al., 2012), which contains matrices constructed from the functional connectome datasets of the “1,000 connectome project” (Biswal et al., 2010). The original datasets in this project were obtained using resting-state fMRI (R-fMRI), which records activation patterns in brain regions during the resting state and is thought to describe the common architecture of the human brain (van den Heuvel et al., 2008; Greicius et al., 2009; Biswal et al., 2010; Van Dijk et al., 2010; Brown et al., 2012). The matrices comprised *N* × *N* elements with *N* = 177 brain regions and were assumed to cover the entire brain. The details of the processing sequence to construct these matrices are shown in the above website and, in this analysis, I use 986 matrices for different individuals, which are available from the same site (Brown et al., 2012).

Brain activity naturally fluctuates and the connectivity matrix contains noise and artifacts (Eguiluz et al., 2005; Fox and Raichle, 2007; Brown et al., 2012). To construct the network structure with significant elements, threshold was applied to the matrix (*w*_*ij*_) (Eguiluz et al., 2005; Brown et al., 2012; Zuo et al., 2012). Because strongly connected pathways form core structures that are relevant to the network structure of the brain (Eguiluz et al., 2005; Brown et al., 2012), I removed connections with small connectivity weights using a threshold and constructed the network with the residual connections. After introducing the threshold *w*_*t*_ for the connectivity weight *w*_*ij*_, I obtained a network description consisting of connections corresponding to the |*w*_*ij*_| > *w*_*t*_ elements. In this analysis, considering the differences between individuals, I defined the threshold value of each individual connectivity matrix *w*_*t*_ based on the average connectivity < |*w*| > and the standard deviation σ_|*w*|_. I calculated < |*w*| > and σ_|*w*|_, and defined the cut-off threshold in terms of the following equation:

(1) $$\begin{array}{l} w_{t}=<|w|>+n\cdot \sigma_{\left|w\right|}, \end{array}$$

with a parameter of *n*.

#### 2.1.2. Structural Connectome Dataset

The simulation results based on the above functional connectome datasets were compared to the structural connectome, and the other connectome datasets describing the physical connection between brain regions. The structural connectome datasets are constructed by the diffusion tensor imaging (DTI) method, which traces the fiber tracts between brain regions and forms another network at the whole brain scale, known as the structural connectome (Sporns et al., 2005). The dataset is available from the above website (http://umcd.humanconnectomeproject.org/) with the pre-processed matrix of the connectivity strength being the same to the fMRI cases (Brown et al., 2012). The DTI dataset is taken from a subsect of the “1,000 connectome project,” tagged as “*NKI*_*Rockland*” for the “Study Name” item, from the Nathan Kline Institute (NKI)/Rockland sample in the web site. It contains the matrices of 196 individuals, and each matrix has *N* = 188 matrix elements (188 × 188).

#### 2.1.3. Information Transfer

Information in the brain is transferred by successive signal propagation; this can be represented by the activated state of each site (Tononi et al., 1994; Hilgetag and Grant, 2000; Beggs and Plenz, 2003; Ghazanfar and Schroeder, 2006; Beggs, 2008; Bressler and Menon, 2010; Sporns, 2013; Takagi, 2018). For each node in the functional network, three states {1, −1, 0} were assigned because the responses of neuronal activity can be categorized as positive and negative (Fox et al., 2005; Shmuel et al., 2006). In this representation, the inactivated regions were assigned the 0 state, while the two states at ±1 represented positive and negative activation states, respectively.

When considering information transfer, I represented a whole state of the brain as *S* = (*s*_1_, …, *s*_*N*_) for a network size *N*, whereby the *i*-th node was assigned as *s*_*i*_ ∈ {1, −1, 0}. I could then calculate the responses *R* = (*r*_1_, …, *r*_*N*_) *r*_*i*_ ∈ {1, −1, 0} for a given connectivity matrix and threshold. For the given set of *S* and connectivity matrix (*w*_*ij*_), the response state was evaluated using the following equation:

(2) $$\begin{array}{l} r_{j}=\sigma \left(\underset{i\in N}{\sum }w_{ij}s_{i}\right). \end{array}$$

I denoted $\sum_{i\in N}w_{ij}s_{i}$ as ${\hat{r}}_{j}$, so a threshold of *w*_*t*_, $\sigma \left({\hat{r}}_{j}\right)$ was defined as *r*_*j*_ = 1, −1, 0 for cases ${\hat{r}}_{j}>w_{t}$, ${\hat{r}}_{j}<-w_{t}$, and $|{\hat{r}}_{j}|\leq w_{t}$. In this simulation, I calculated the information transfer of stimuli *S*. The input signals were taken randomly, although I did use the same probability for positive and negative activation. I then assigned 1 and −1 to each input signal *s*_*i*_, with the probability *p* being set to 0 in the other cases with the probability 1−2*p*. Each condition in this simulation was repeated 100 times with each input signal.

### 2.2. Statistical Quantities of Information Transfer Model

#### 2.2.1. Mutual Information

To measure information transfer from the imposed stimuli to the responses, I evaluated the mutual information for the set of stimulus signals *S* and the corresponding responses *R*. It is defined as *H*(*R*) − *H*(*R*|*S*) with *H*(*R*), the information of the response *R*, and *H*(*R*|*S*), the conditional entropy. This quantity was used to assess the efficiency of information transfer in the neural network models and in real biological data (Beggs and Plenz, 2003; Beggs, 2008).

In the analysis, the mutual information of the transfer between *i* and *j* nodes was estimated using the following equation:

(3) $$\begin{array}{l} m\left(i,j\right)=H\left(s_{i}\right)+H\left(r_{j}\right)-H\left(s_{i},r_{j}\right), \end{array}$$

where the entropy *H*(*s*_*i*_) and *H*(*r*_*j*_), as well as the joint entropy *H*(*s*_*i*_, *r*_*j*_), were calculated using the probabilities of each state: *s*_*i*_, *r*_*j*_ ∈ {±1, 0}. Next, this quantity was estimated for the whole network as follows: $m=\sum_{j}<m(j))>\;/N$, with averaging as $<m\left(j\right)>=\left(\sum_{i}m\left(i,j\right)\right)/\left(N-1\right)$ for all possible connections of each node *j*.

#### 2.2.2. Network Energy

The energy of the brain network is described in different ways, which are mainly categorized into wiring costs for organizing the network structure and those related to their activity. The total number of connections determine the wiring cost to organize the network structure (Achard and Bullmore, 2006; Bullmore and Sporns, 2012). Thus, the wiring cost based on the topological structure basically describes the energy demands of the brain functional network. It is assumed that many characteristic attributes of the brain network can be explained by minimizing the wiring cost (Bullmore and Sporns, 2012).

Hence, I defined this energy, the wiring cost denoted as *E*_*W*_, using the following equation:

(4) $$\begin{array}{l} E_{W}=\underset{i,j}{\sum }a_{i,j}, \end{array}$$

where *a*_*i, j*_ denotes the element of the adjacency matrix. For an undirected topological graph of a given matrix, the connection for each pair of *i* and *j* was represented using the adjacency matrix element, which is connected as *a*_*i, j*_ = 1 for |*w*_*ij*_| > *w*_*t*_, with threshold *w*_*t*_, and disconnected as *a*_*i, j*_ = 0 in other cases.

On the other hand, the Hopfield energy gives a definition related to the dynamics and the associated information of the neural networks. For a given network state of activation, the Hopfield energy provides one definition of the network energy. It models the network state of the neurons and can also be applied to artificial neuronal networks (Hopfield, 1984; Hinton and Salakhutdinov, 2006). Hopfield networks and similar types of energy representation have been introduced to describe the energy state of neural networks, modeling the spin glass network (Hopfield, 1984). One example of the artificial learning models that use this type of function is the restricted Boltzmann machine, which evolves by adjusting the network variables according to rules learned from the energy function (Hinton and Salakhutdinov, 2006). It is defined as

(5) $$\begin{array}{l} E_{H}=-\left(\underset{i,j}{\sum }r_{i}w_{i,j}r_{j}\right) \end{array}$$

whereby I took a bias-free case in accordance with the transfer model Equation (2).

In the original definition of the Hopfield energy (Hopfield, 1984) bias terms are present, such as those expressed as $\sum_{i}r_{i}b_{i}$ with constant bias *b*_*i*_ assigned the value for each node. In this simulation, they are excluded as the constants under the assumption of homogeneity of nodes. According to this simplification, the simulation is given in the bias-free form, and takes 0 for the cases, such as the random state as well as negative values, especially in the low energy states.

However, as the indicator of total activity cost, the Hopfield energy would be estimated as small as the positive and negative terms were not included in the definition. To avoid this cancelation and estimate the total energy cost for the activity, I introduced a definition of the activity cost using the absolute values of each term and compared them with the values mentioned above Equation (5). The definition given was as follows:

(6) $$\begin{array}{l} E_{A}=\underset{i,j}{\sum }|r_{i}w_{i,j}r_{j}| \end{array}$$

represents the total energy cost for the activation dynamics. It assesses the contributions from the positive and the negative signal equally and then evaluates the total amount of signal activations with their weights. In the discussion, I assessed the energy of the functional brain network based on these definitions.

#### 2.2.3. Overlapping Number in the Activation Patterns

I analyzed the pattern of the response signals *R* = (*r*_*i*_) using the overlapping numbers of the different signals. I evaluated it in terms of the number of regions activated or deactivated equally with the different patterns. For the set of response patterns $R^{j}=\left(r_{i}^{j}\right)$, whereby *j* is an index of the input state, I counted the number of the same responses $r_{i}^{j}=r_{i}^{j^{\prime }}$ for the pair *j* and *j*′. I normalized this overlapping number by the total number of regions *N*. I then wrote it down as

(7) $$\begin{array}{l} h\left(j,j^{\prime }\right)=\underset{i}{\sum }\pi \left(r_{i}^{j},r_{i}^{j^{\prime }}\right)/N \end{array}$$

where $\pi \left(r_{i}^{j},r_{i}^{j^{\prime }}\right)$ is 1 for $r_{i}^{j}=r_{i}^{j^{\prime }}$ and 0 was taken in the other cases. I then took the averages of all the pairs of *R*^*j*^ and *R*^*j*^′.

The definition of the overlapping number (Equation 7) is the same as that of the Hamming distance of the information theory. It is used, for example, to measure the error in the signal transfer. In the analysis, it was used to analyze the relationship between the activation patterns and efficiency of the information transfer. As excess overlapped states indicated that the variation in the response *S* is lost, they resulted in the decrease in the mutual information entropy.

The program for this network model is available at https://github.com/coutakagi/fcn2019.git.

### 2.3. Network Structure and Statistical Evaluation

The functional connectome is often described in topological or weighted terms. Different measures are required to assess the topological network structure, especially in terms of criticality. To specify the criticality in the activation dynamics, the characterization is given by the statistics of the avalanche events. One measure is the mutual information entropy, such as defined above, which is maximized in this state in comparison to the super-critical state (in which excess activation is saturated) and the sub-critical state (in which activations die out due to poor sensitivity to the stimulus) (Beggs and Plenz, 2003; Beggs, 2008). This is contrasted to the criticality of the topological structure, which is usually characterized by appearances of the giant connected component or other states, such as the small-world topology (Watts and Strogatz, 1998), which are evaluated by quantities, such as degree or the clustering coefficients.

Besides the total number of connections, topological structures were measured in terms of the largest connected component to provide a basic measure of the topological network. With using the adjacency matrix, the size of each connected component was then measured in terms of the number of nodes in each connected subgraph, and these values determined the largest connected component of each network. In the present paper, I measured this quantity using R-package igraph (Barrat et al., 2004).

On the other hand, to account for connectivity strength *w*_*ij*_, I took the absolute node strength value $ns_{i}=\sum_{j}|w_{ij}|$ in each node and evaluated its statistical features using a distribution model (Takagi, 2017, 2018). Due to the criticality of the brain (Achard et al., 2006; Bassett and Bullmore, 2006; Hagmann et al., 2008; van den Heuvel and Sporns, 2011; Takagi, 2017, 2018), the distributions of network variables, such as degree, exhibit a characteristic shape similar to the power law. However, when I adapted the power law to the distributions, this straightforward application was prohibited because the energy constraints on brain activity constitute an upper limits (Takagi, 2017, 2018). In the present study, the same assumption was applied, and I introduced an upper strength limit of *ns*_*max*_. Following this assumption, I obtained an expression for the normalized variable $\tilde{s}=\;\left(ns_{max}-ns\right)$ as

(8) $$\begin{array}{l} p\left(ns\right)\propto {\left(\tilde{n}s\right)}^{\gamma }={\left(ns_{max}-ns\right)}^{\gamma }, \end{array}$$

with a constant γ (Takagi, 2017, 2018).

Next, I assessed the strength distribution *ns* in terms of deviations from this model using the Kolmogorov-Smirnov (KS) distance (Clauset et al., 2009; Klaus et al., 2011). For the cumulative distribution *p*_*e*_(*ns*), which was experimentally given, and that of the model *p*_*c*_(*ns*), which was fitted to the data, the KS distance *D* was defined using the following equation:

(9) $$\begin{array}{l} D=\underset{w}{\max}|p_{e}\left(ns\right)-p_{c}\left(ns\right)| \end{array}$$

which measures the maximum distance of the model from the experimental data. If this value was sufficiently small, the network probably exhibits the feature characterized by this distribution model.

Finally, I measured the clustering coefficient *C*, also known as transitivity, for each adjacency matrix. This is another important topological quantity which is often used as an indicator of the small-world network (Watts and Strogatz, 1998). It is defined as the probability that the adjacent vertices of a vertex are connected (Watts and Strogatz, 1998). Here, it is measured for each adjacency matrix, using the R-package igraph (Barrat et al., 2004).

## 3. Results

### 3.1. Information Transfer Model

**Figure 1:**
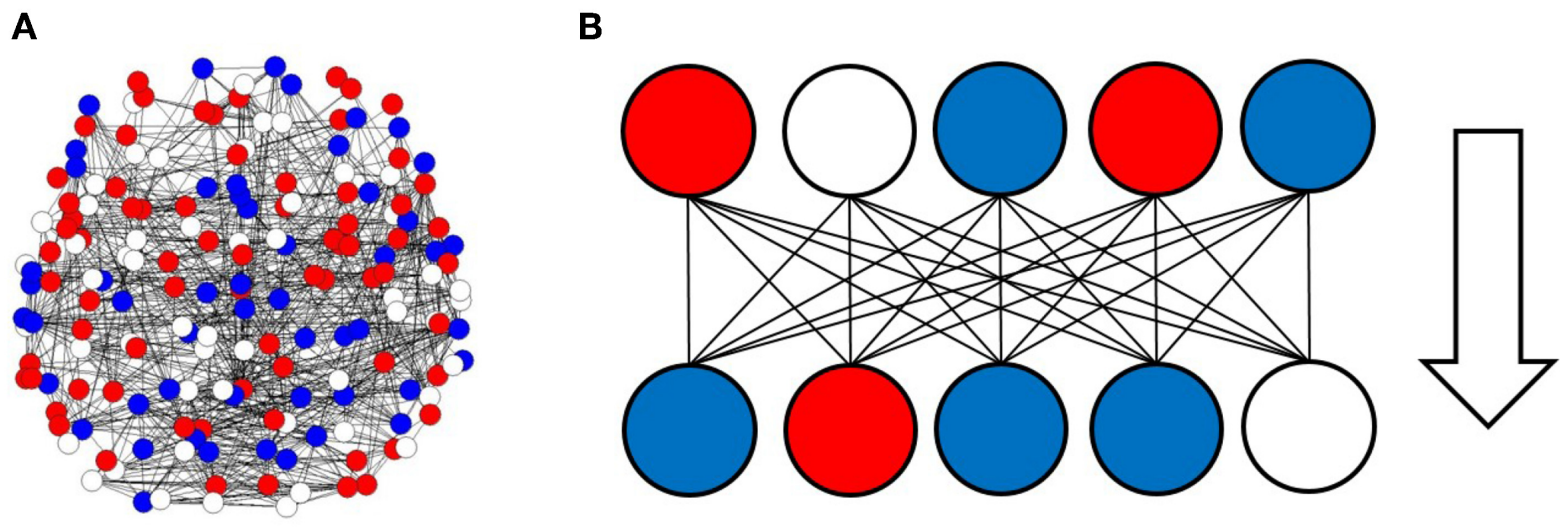
Activation and deactivation patterns in the local regions, and the information transfer associated with these patterns. **(A)** Activation and deactivation in the brain is illustrated. In this figure, each circle represents the states of local regions in the brain, with solid lines corresponding to the connection between regions. Activated regions are represented by red circles, while negatively activated ones are colored in blue. The residual white circles correspond to other inactivated states. **(B)** The information transfer associated with these patterns is illustrated. The pattern state in the upper side, which is shown on the line, consists of signals transferred to the lower sides, where each region state is changed according to the upper input patterns and the connection strengths between the regions.

To analyze the information processing in the large scale network of the human brain, I simulated information transfer using successive activation patterns. Because activity in the brain can be observed as activation and deactivation in local regions, signal transmission associated with information processing can be described in terms of successive changing at each site, with positive or negative activation (Beggs and Plenz, 2003; Fox et al., 2005; Ghazanfar and Schroeder, 2006; Shmuel et al., 2006; Beggs, 2008; Bressler and Menon, 2010; Takagi, 2018). In the model, the given brain state sites, as illustrated in Figure 1A, were transferred to successive states, which were determined by the correlation among the sites given by the matrix (*w*_*ij*_) as Figure 1B.

**Figure 2:**
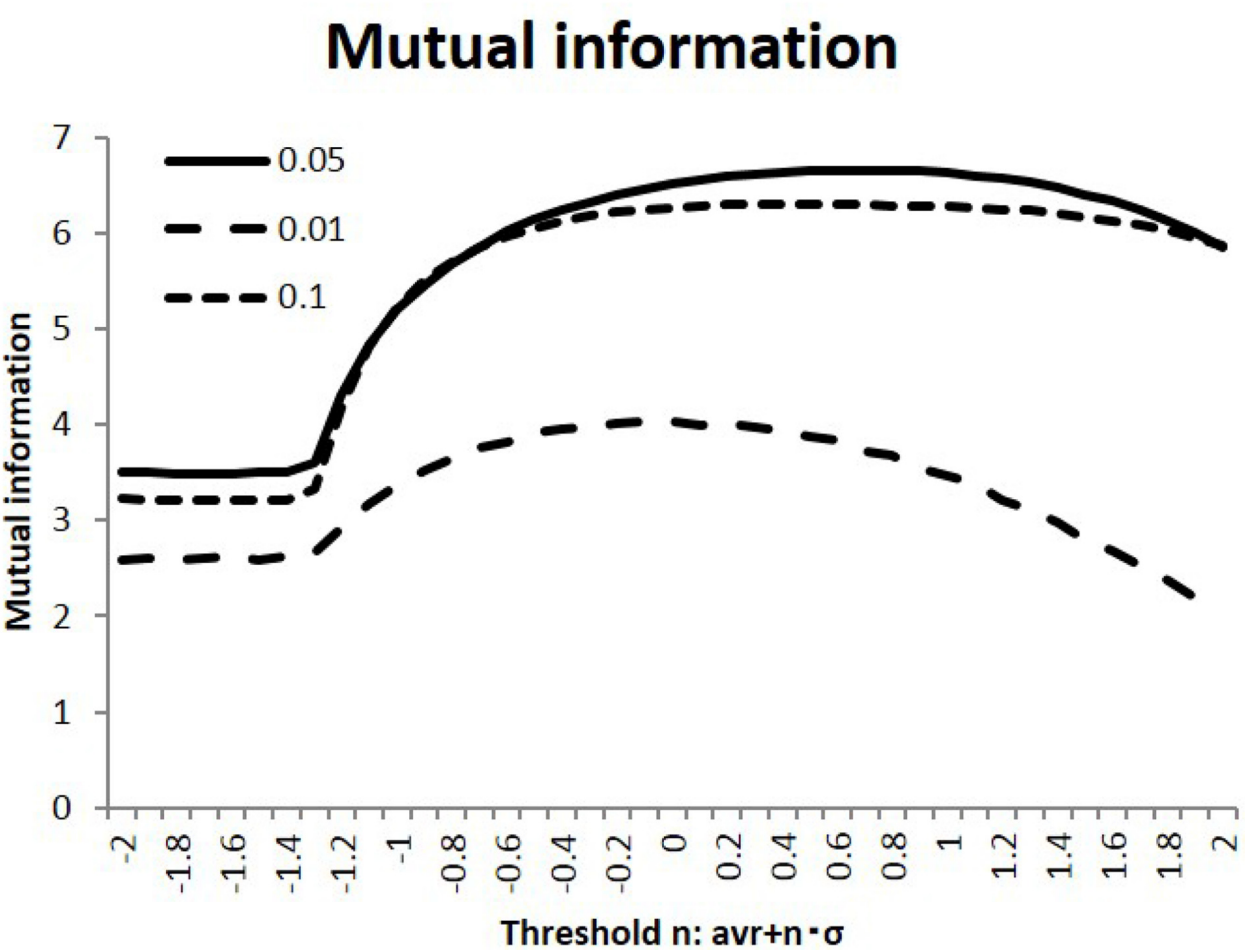
Mutual information with different activation probabilities. Three different values of the activation probability are shown: *p* = 0.1, 0.05, 0.01. These conditions are shown with the dotted, the dashed, and solid lines, respectively. The different states are measured, with the threshold values on the x-axis being varied. I took the threshold as < |*w*| > +*n*·σ_|*w*|_ with the connectivity average < |*w*| > and its standard deviation σ_|*w*|_ for each connectivity matrix. Following this, the mutual information is evaluated by taking the average of Equation (3).

In the simulation, I calculated the response state *R* = (*r*_*i*_) of the randomly stimulated signals *S* = (*s*_*j*_), as represented by Equation (2), using the connectivity matrices (*w*_*ij*_) of the human connectome. Next, as shown in Figure 2, I evaluated the efficiency of transfer of the mutual information, defined as the average of Equation (3). As part of the preliminary evaluation, I used randomly selected 100 individual matrices for calculation. I compared this quantity among the different states, which were varied in terms of the connectivity strength threshold value *w*_*t*_ and the activation probability of the input stimuli. As shown in this figure, information transfer depended on these parameters, while the activation probability *p* = 0.05 gave the maximum values for these different conditions. Starting from the flatten values for lower thresholds due to its negative threshold value on the left end, the measurements of the mutual information entropy increased to their maximum values in the intermediate states. Moreover, the standard deviations for the thresholds *n* = 1.0, 0, −1.0 were evaluated for *p* = 0.05 as 9.11 × 10^−2^, 7.45 × 10^−2^, 1.34 × 10^−1^. These values were smaller than their mean values, and these results were stable. Because I were interested in the state with maximum mutual information, I used this value, *p* = 0.05, in the following simulation.

### 3.2. Network Energy and Efficiency of the Information Transfer

**Figure 3:**
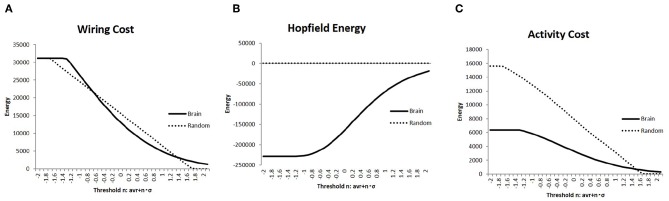
The three different types of energies, the wiring cost (Equation 4), the Hopfield energy (Equation 5), and the activity cost (Equation 6) are shown in **(A–C)**, respectively, with the solid lines on each graph. They are compared with the results of the random network, which are shown in the dashed lines. I applied the same threshold set of values to Figure 2.

Constraints regarding energy would be a major factor regulating network structure and activity in the brain (Bullmore and Sporns, 2012). Hence, I evaluated the network energy of each brain state, which is a basic parameter to analyze brain activity. Then, I showed the results of the measured energies using three different definitions in Figure 3; in each graph, the connectivity strength threshold differed. Further, I compared these values with those of the random networks, which were considered as the null model. The random networks with the same network size were determined together with the randomly taken weights *w*_*i, j*_ ∈ [−1, 1], and 1,000 random matrices were obtained. On the contrary, the results for the brain network were measured using the whole datasets, which contained 986 matrices of different individuals.

**Figure 4:**
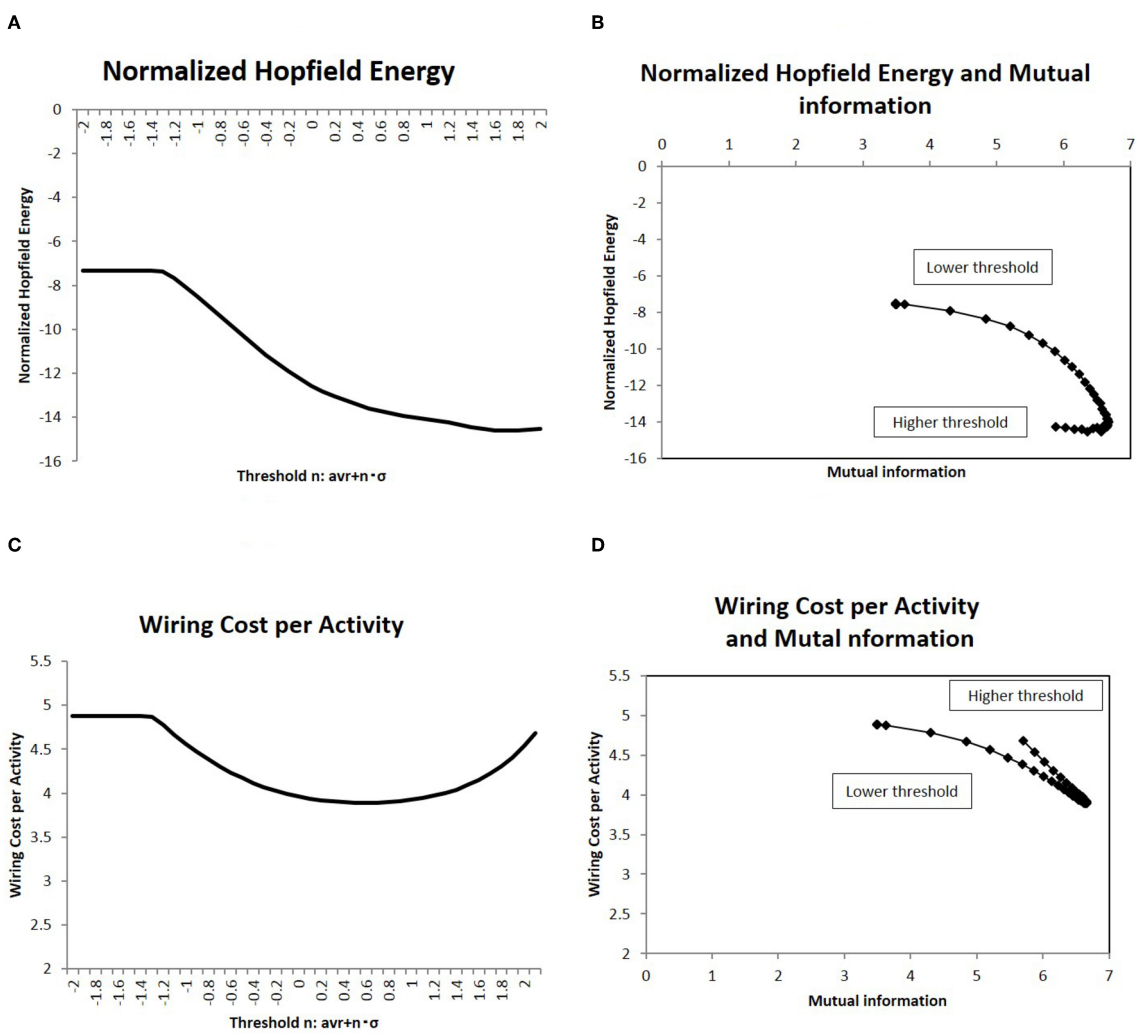
Network energy and the correlation with mutual information. **(A)** The Hopfield energy *E*_*H*_ defined in Equation (5) is normalized by wiring cost *E*_*W*_, Equation (4), and this normalized energy *E*_*H*_/*E*_*W*_ is shown for the different threshold values. I applied the same threshold set of values to Figure 2. **(B)** showed a correlation between mutual information and the normalized Hopfield energy. On the x-axis, I took the mutual information calculated using the whole datasets of matrices by applying the method, which is denoted by a solid line (*p* = 0.05) (Figure 2). I plotted the corresponding points of each threshold value with the network energy on the y-axis, as given in **(A)**. **(C)** Another normalized energy, *E*_*W*_/*E*_*A*_ where the wiring cost *E*_*W*_ is defined as Equation (4) and the activity cost *E*_*A*_ is defined as Equation (6), is shown. The threshold is the same as that of the case of **(A)**. Its correlation with mutual information entropy on **(D)** is the same as the case of **(B)**.

The wiring cost defined in Equation (4) is shown in Figure 4A. It was compared with the wiring cost of the random network, which appeared as a straight line proportional to *n* of the threshold value defined in Equation (1). In comparison with these networks, it was found that the plotted curve of the brain network has a relatively long tail for higher values of *w*_*t*_, which indicated the well-known attributes of the brain network, such as the scale-free and small-world network, as will be discussed.

The difference between the brain network and the random one was enhanced in comparison with the values of the Hopfield energy. Figure 4B shows the relatively large values for the brain, while it took almost 0 for the random network due to the cancellation of the positive and negative terms. To avoid this cancellation and evaluate the total amount of the activity cost, I calculated the energy with another definition given in Equation (6) and plotted it in Figure 3C. The energy for each range except for 0 states had higher *w*_*t*_ values; the activity cost for the random model was higher than those of the brain network as expected.

### 3.3. Normalized Energy and the Mutual Information Entropy

Due to energy constraints, it was assumed that the activities for the information transfer is required to be efficient (Bullmore and Sporns, 2012). One description of the network efficiency for a given cost was based on the energy consumed during the activity, which was normalized by the wiring cost to organize the network structure (Takagi, 2017). Then, at first, the Hopfield energy was normalized with the wiring cost as *E*_*H*_/*E*_*W*_ and shown in Figure 4A. With regards to mutual information, the correlations are depicted in Figure 4B, which indicates a negative correlation, whereby decreasing the network energy resulted in increases in mutual information. The same figure shows that there was a peak around the maximum point of mutual information, where mutual information was maximized and the network energy associated with activity was minimized.

To clarify the cost performance of the activity in the brain, I took another quantity, *E*_*W*_/*E*_*A*_, the wiring cost (Equation 4) normalized by the activity cost (Equation 6). This normalized quantity represents the wiring cost required to maintain a unit amount of activity. The measurement is then shown in Figure 4C, and its correlation with the mutual information entropy is presented in Figure 4D. It shows the clear correlation with a sharp peak, around which the mutual information is maximized and the normalized wiring cost is minimized. These results (Figures 4B,D) for different definitions of the normalized energy exhibit the similar behavior and the clear dependency of the mutual information entropy on the network energy. Thus, these peaks on correlations define the optimal state of the brain functional network, in which the efficiency of information transfer for a given network energy cost was maximized.

### 3.4. Network Structure and the Optimal State

**Figure 5:**
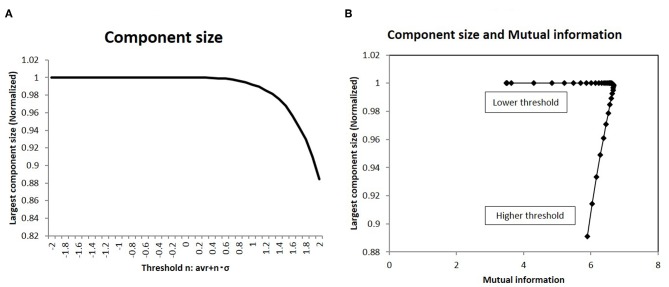
The largest component size of the functional network and the mutual information **(A)**. I showed the largest component size of the topological representation for each threshold values. I took the average of the values of the largest component size, which are normalized by the total number of nodes in the topological graph. **(B)** I show the relation between the mutual information and the largest component size. On the x-axis, I took the mutual information calculated using the whole datasets of matrices by applying the method, which is denoted by a solid line (*p* = 0.05) (Figure 2). Then I plot the corresponding points for each threshold value with the largest component size on the y-axis, which are given in **(A)**.

I analyzed the network structure around this peak state. At first, the topological network structure of each state around this point was characterized in terms of the largest component size: a basic quantity of the network topology. This result is shown in Figure 5A, wherein the component size is shown normalized to the network size. In the same graph, the largest component size of the connected subgraph decreases with increasing threshold, with the normalized size being 1, which corresponds to the fully connected graph. Next, I took the correlation between mutual information and this quantity in Figure 5B. The sharp peak on this graph indicates that maximum information was realized in the fully connected graph with minimum connections.

**Figure 6:**
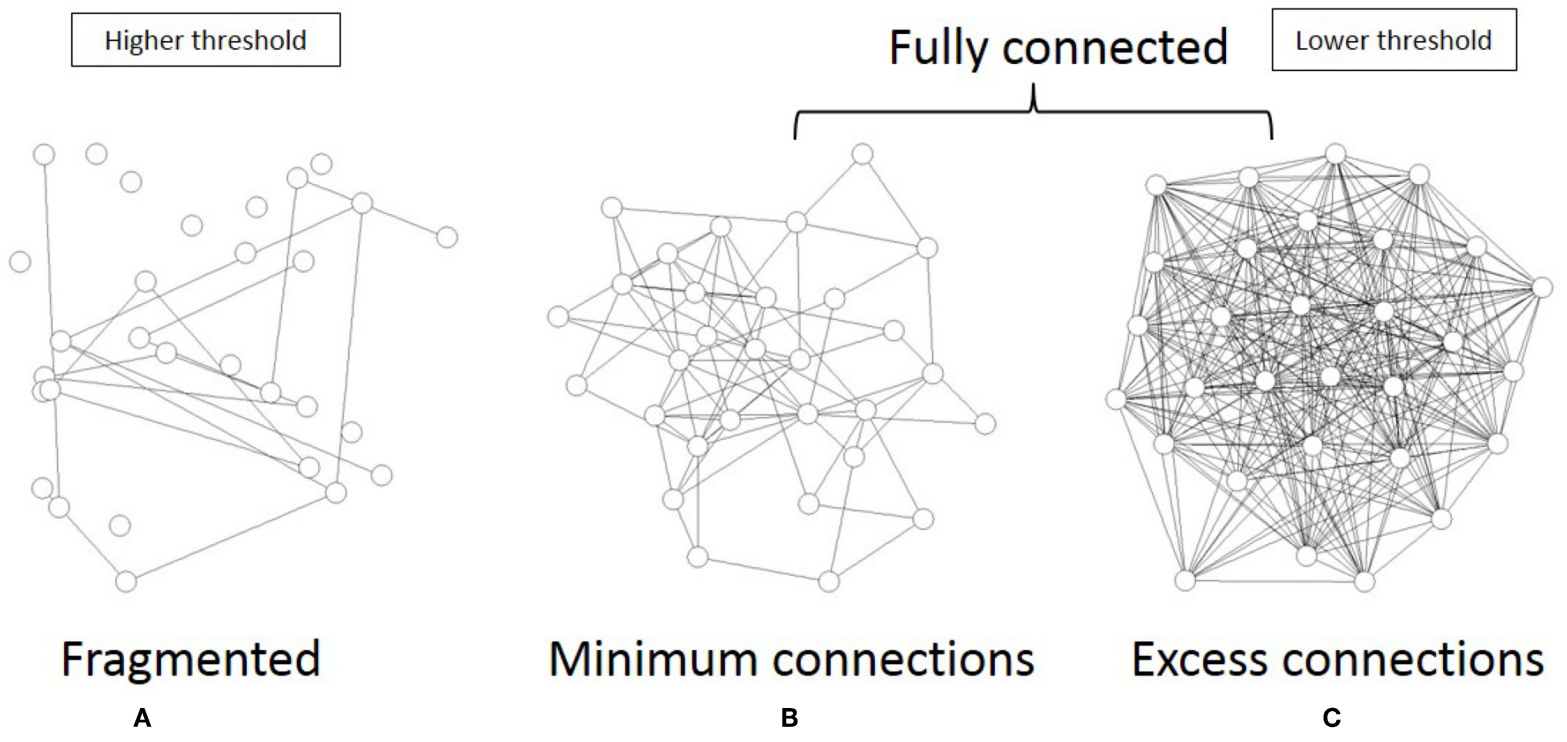
I illustrate the three different states of the topological network structures. **(A)** I show an example of the fragmented sate which contains the disconnected components. **(B)** I show the critical state of the network topology, all components of which are fully connected with the minimum connections. **(C)** This state is also the fully connected network, but it contains excess connections.

As shown in Figure 6, the topological network graph contains excess connections in the lower threshold. In this state, signals with information transfer also contain noise due to these excess elements. At the higher threshold value, the network loses this fully connected structure, and the graph is fragmented into multiple disconnected sub-components, as shown in Figure 6A. In this state, mutual communication between disconnected nodes is hindered, so the efficiency of the information transfer might be reduced. The sharp peak on Figure 5B corresponds to the boundary state between these two states, where the network preserves the fully connected structure with minimum connections. Combined with the correlation between mutual information and network energy (Figures 4B,D, 5B), this result can be interpreted as showing that efficiency and energy consumption are optimized in this state, with a fully connected structure that eliminates transfer noise.

**Figure 7:**
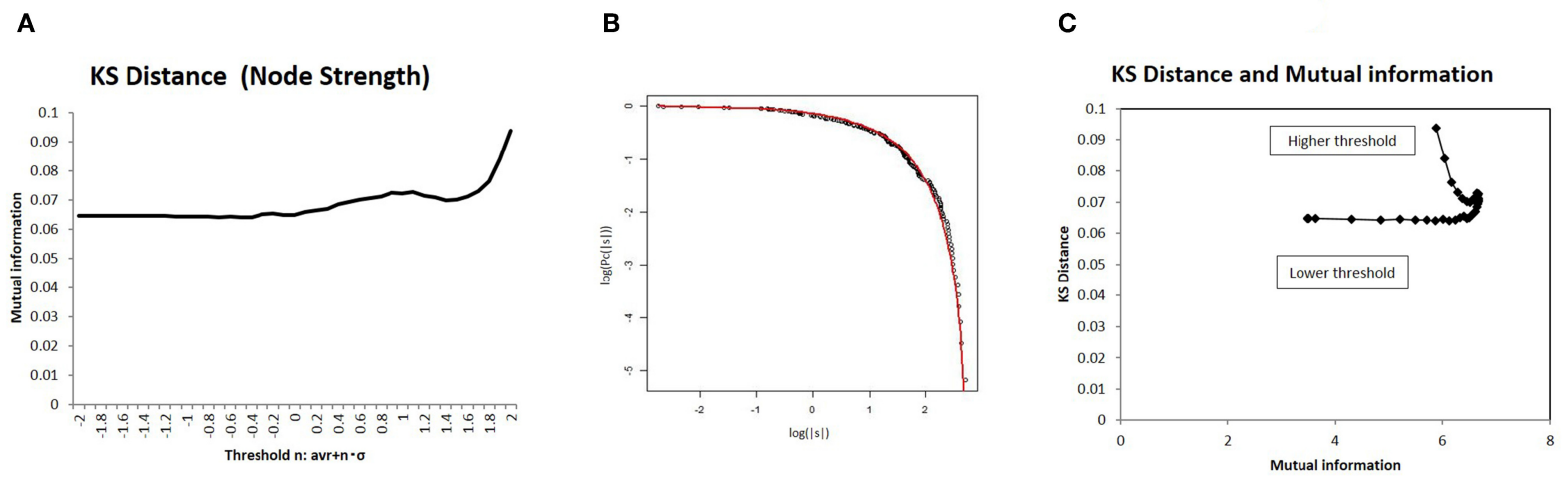
I evaluated the distribution of node strength in terms of deviation from the distribution model using Kolmogorov-Smirnov (KS) distance. I showed a correlation with mutual information. **(A)** I also showed KS distance values from the distribution model for different threshold values. I assessed this value in terms of node strength, as well as the sum of the absolute values of connectivity strength for each node, using the distribution model. The threshold values were taken as the same as in the other cases in Figure 2. **(B)** Using an example, I compared empirical distribution and the distribution model. The distribution was taken as the threshold < |*w*| > with *n* = 0, at which the average KS distance was evaluated as 6.64 × 10^−2^. **(C)** I showed a correlation between mutual information and KS distance. On the x-axis, I took the mutual information calculated using whole datasets of matrices by applying the method, which is denoted by a solid line (*p* = 0.05) (Figure 2). I then plotted the corresponding points for each threshold value with KS distance on the y-axis, as given in **(A)**.

Because this optimal state resides in the boundary state between the fully connected and fragmented phases, it constitutes a critical state, whereby connectivity strength, another important variable of network structure, shows a characteristic distribution. To introduce a distribution model for this critical state (Equation 8), I measured the statistical deviation of the total connectivity strength of each node *ns* in Figure 7 using the KS distance, defined as Equation (9). As shown in Figure 7B, the KS distance measured about 0.07 around its minimum value, which was a sufficiently small fitting. Moreover, the model fitting was validated by comparison to other distribution models (Takagi, 2017, 2018). In the case of node strength, the KS distance values of this model 6.6 × 10^−2^ and of the normal distribution 8.5 × 10^−2^ support this model, with its lower value. In addition, the correlation with mutual information is shown in Figure 7C, which indicates that the characteristic distribution of *ns* depends on this quantity, as is the case with larger component sizes (Figure 5B) and with energy (Figures 4B,D). Therefore, around the optimal state defined for efficiency and energy, the distribution of the node strength converges on this model, and the characteristic network structure emerges.

### 3.5. Activation Patterns and Overlapping

**Figure 8:**
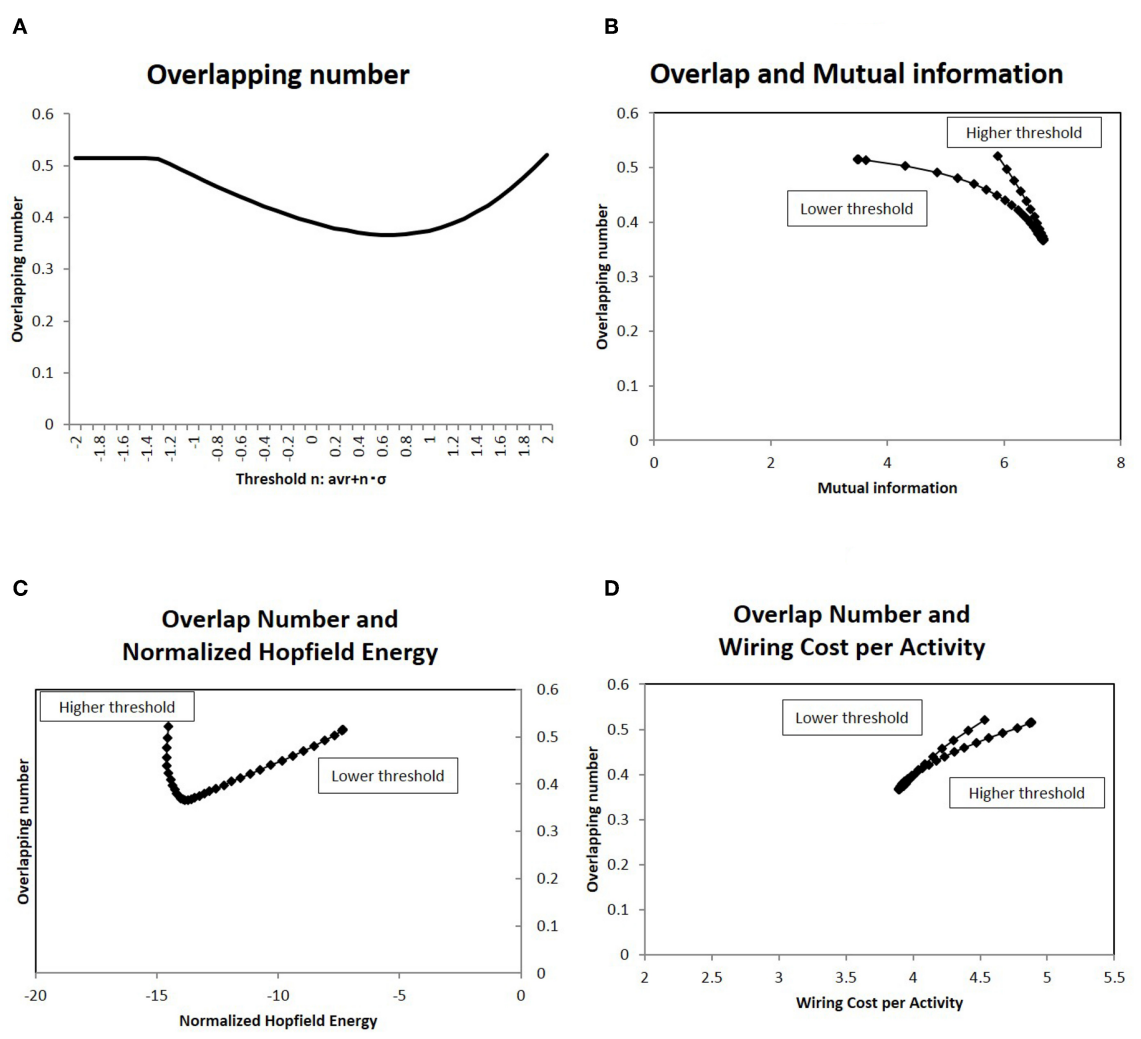
I show the number of overlapping patterns in the response signals, as well as their correlation with the mutual information **(A)**. I showed the average of the number of overlapping co-activated regions defined as Equation (7). I took different network states by varying the threshold, as with the cases in the other figures. **(B)** I showed the correlation between the overlapping patterns given in **(A)** with the mutual information. On the x-axis, I took the mutual information calculated using the whole datasets of matrices by applying the method, which is denoted by a solid line (*p* = 0.05) (Figure 2). Then I plotted the corresponding points of each threshold value by overlapping numbers on the y-axis, which are given in **(A)**. **(C)** There was a correlation between the overlapping patterns given in **(A)** and the normalized Hopfield energy. On the x-axis, I measured the normalized Hopfield energy given in Figure 5A. I plotted the corresponding points of each threshold value with the network energy on the y-axis, as given in **(A)**. **(D)** A correlation was observed between the overlapping patterns given in **(A)** and the wiring cost performance given in Figure 5C, similar to **(C)**.

To analyze how this characteristic state regulates information transfer, I investigated the overlapping patterns of the response signals, applying the results of the information transfer model to Equation (2). As explained above, the repeatedly co-activated regions of different stimulation signals are related to cognitive processes (Haxby et al., 2001; Kumaran et al., 2016). For the set of response signals to random stimuli, the number of overlapping co-activated regions between different response signals was quantified in terms of Equation (7). The results for different network states are shown in Figure 8A, where the average of the overlapping numbers is taken for all combinations of responses. From lower thresholds to higher ones, the overlapping number decreased with decreasing excess connections. While it took large values at higher thresholds, it took the minimum value at the intermediate state.

The correlation to efficiency of information transfer is shown in the next panel (Figure 8B), in which the mutual information and the number of the overlapping sites has a strong correlation. As indicated by this graph, the overlapping number took the minimum value for maximum mutual information. On the other hand, the same number reduced network energy, as shown in Figures 8C,D, which show negative correlation. Therefore, the activation patterns evaluated in terms of the overlapping numbers are correlated strongly with the statistical quantities, network efficiency and energy.

**Figure 9:**
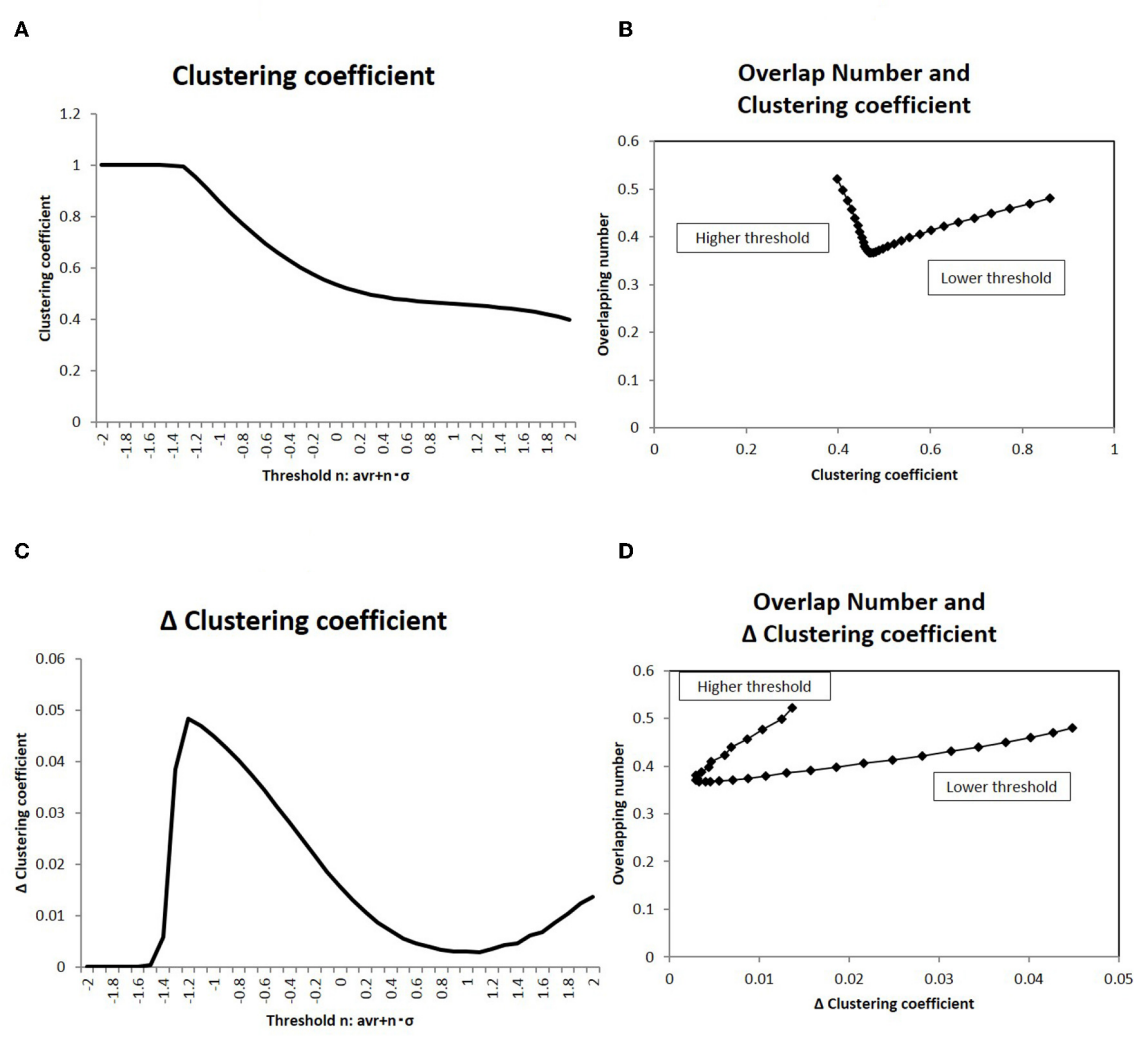
Clustering coefficient and overlapping number. **(A)** The clustering coefficient *C* for the topological description with the adjacency matrix is averaged and shown. The threshold values in the x-axis and the corresponding adjacency matrices are taken to be the same as those in Figure 4. The datasets of the matrix are also the same to those used in Figure 4. **(B)** The correlation of the clustering coefficient *C* (shown in **A**) to the overlapping number is shown. The overlapping number is the same to those in Figure 8A. The threshold range in this panel is taken in [−1.0, −2.0] so as to exclude the flat values in the lower thresholds. **(C)** The differences of the clustering coefficient in **(A)** is shown. The difference Δ*C* is calculated as Δ*C* = *C*(*i*) − *C*(*i* + 1), where the difference is taken with the next value in the graph and *i* is the number of the threshold position counted from the lower side. **(D)** The correlation of the clustering coefficient difference Δ*C* (shown in **C**) to the overlapping number is shown. The values of the overlapping numbers are the same as those in Figure 8A. The threshold range (which was the same as **C**) is taken.

The relation between the overlapping number and the network topological structures were also analyzed in Figure 9, which shows the direct relation to the small world topology. As described in the introduction, the small-world structure is considered as another relevant attribute of the brain network. The clustering coefficient was measured for each threshold value in Figure 9A. The correlation to the overlapping number was plotted on Figure 9B, in which a sharp peak around the minimum overlapping number indicated that the phase transition occurs around this point (with respect to the topological structure). The further evidence for the relation to small-world topology is given by the changes of this value. According to the observation in the Watts-Strogatz model (Watts and Strogatz, 1998), the clustering coefficient is stable near the state of the small-world topology, which is accompanied by the phase transition. The changes to the clustering coefficient *C* were taken as the difference from the neighbor value, and were plotted in Figure 9C (Takagi, 2018). The correlation against the corresponding overlapping number is shown in Figure 9D. This result explicitly shows the dependency of the stability of the clustering coefficient and the phase transition of the topological structure. Thus, the minimization of the overlapping number can be correlated to the small-world topology.

### 3.6. Comparison to the Different Datasets

In order to verify the robustness of the above results, the simulation results based on other matrix datasets are presented. The first set is the sub-matrix, which is taken with randomly selected nodes from the original matrix of the functional connectome. The other set is the structural connectome, which is constructed using the physical connections of fiber tracts in the brain with the DTI method.

**Figure 10:**
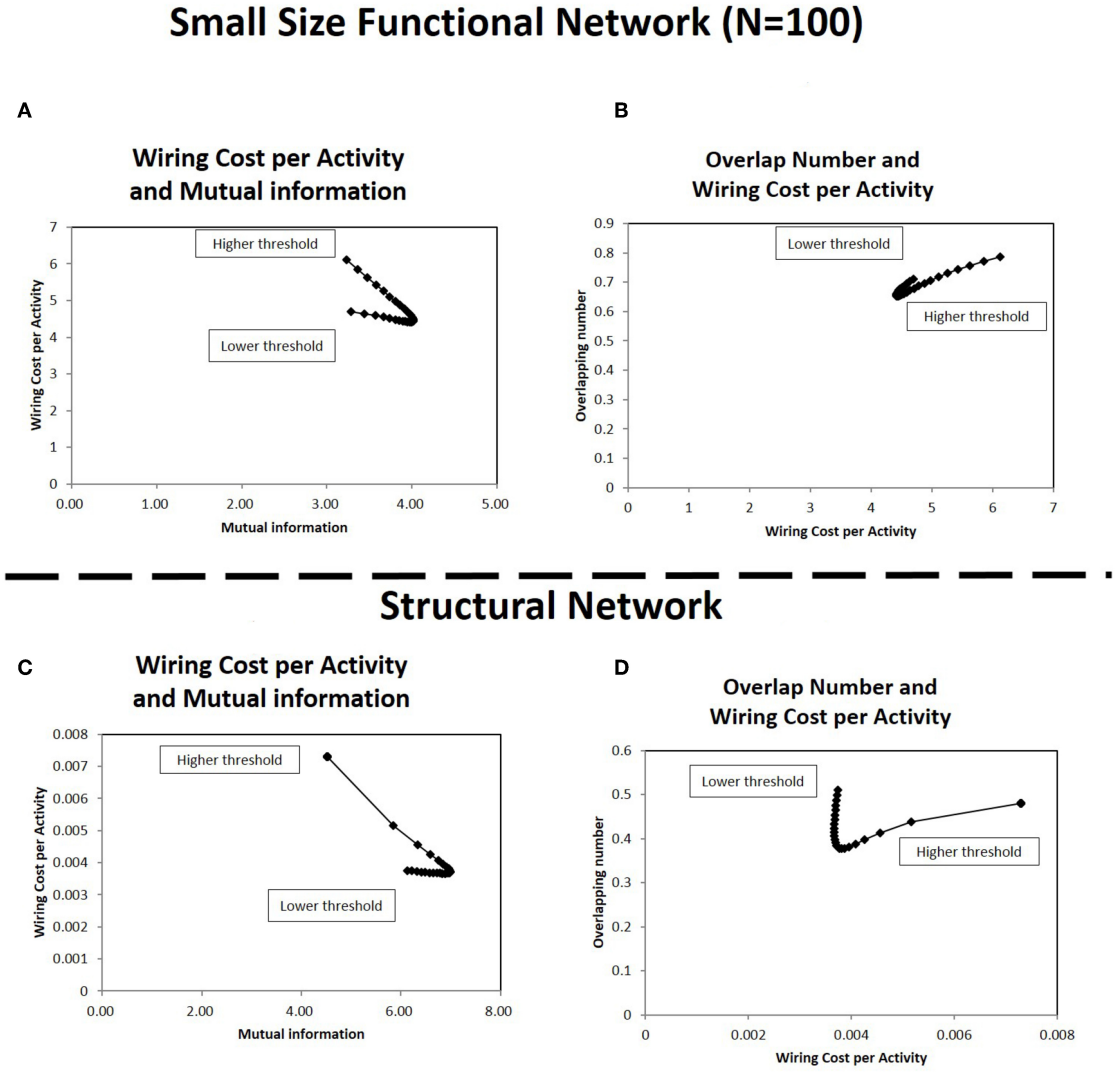
Correlations in the small size functional network and the structural network. In **(A,B)**, the correlation between the quantities shown in Figures 4D, 8D are estimated, respectively for the small size functional network. The small size network, 100 × 100 matrix, is taken from the original 177 × 177 matrix with randomly selected 100 nodes. The calculation methods for each panel is same to corresponding ones in each figure. The threshold range in this panel is taken in [−1.0, −2.0], the same as in Figure 9 to exclude lower threshold ranges which are almost flat. In panels **(C,D)**, the correlation between the same quantities are estimated for the structural network. The connectivity matrix constructed from DTI images are downloaded from the same website as those of the functional connectome dataset (http://umcd.humanconnectomeproject.org/) (Brown et al., 2012). The evaluation methods are the same as those for the above panels **(A,B)**.

At first, the results with the sub-matrix were analyzed (Figures 10A,B). This simulation uses the connectivity matrices size in 100 nodes, which are selected randomly from the total 177 nodes in each original matrix. Comparison of Figures 4D, 10A shows the relations between the wiring cost and the mutual information, and the minimization/maximization relations between these quantities are exhibited adequately in these panels. The other relation (Figure 8D) is also supported by Figure 10B, which displays the relation between the overlapping numbers and the wiring cost. These results with the sub-matrices show that important properties between the overlapping numbers and the wiring cost are stably obtained. The results suggest that these values are independent to other factors, such as the connectivity matrix size or the specific location of the brain regions taken as nodes.

The results with the structural connectome are displayed in Figures 10C,D. The results observed in the functional connectome can be confirmed with Figures 10C,D, where the simulation results exhibit similar properties to those given with the fMRI datasets (Figures 4D, 8D), respectively. They also agree with the similarity between the functional and the structural connectome, in that the functional connectivity in the restring-state has close relation to the physical connections, such as the fiber tracts which organize the structural connectivity (Biswal et al., 2010). Thus, the robustness and the stability of the major properties obtained in this paper are given more strong evidence by the results of the structural network datasets. Because the structural network is comprised of the fiber tracts, the network structure is more stable compared to the functional connectivity based on the temporal dynamics correlations. In addition, the results obtained with the physical connections further clarify the meaning of the energy. In particular, wiring cost can be explicitly related to the real energy cost of the brain for network formation.

## 4. Discussion

In the present paper, I modeled information transfer in the brain based on a dataset of the human functional connectome. As illustrated by Figure 1A, I represented brain activity using the activation patterns of multiple regions. That is, information processing was modeled in terms of the dynamics of successive patterns of activation. These dynamics were described in terms of the changing of activation states, as illustrated in Figure 1B, wherein positively or negatively activated states were transferred by activating or inactivating connected regions.

### 4.1. Information Transfer Model and Basic Statistical Quantities

In this simulation, I calculated the information transfer of randomly activated signals using Equation (2). Using this model, I evaluated the mutual information, defined as the average of Equation (3), and the network energy, defined as Equations (4–6). They are shown in Figures 2, 4A, respectively. On the other hand, numerous empirical studies have suggested that information transfer in the brain is optimized, under constraints, such as the energy consumption, by maximizing mutual information in the communication between brain regions (Linsker, 1990; Friston, 2010; Bullmore and Sporns, 2012). Therefore, I assessed the correlation between these two quantities. In these results given in Figures 4B,D, the energy is evaluated in terms of its cost performance, then the Hopfield energy normalized by the wiring cost and the wiring cost per total activity cost are shown, respectively and the decreasing of these quantities indicates the improvement of the cost performance. I showed these relationships in Figures 4B,D, in which I plotted the corresponding values of each network state. The figure indicated a negative correlation between the values, whereby increases in mutual information led to decreases in network energy, and vice versa. Thus, these two quantities must be correlated.

In particular, the peak around the maximum mutual information in Figures 4B,D shows that information transfer is optimized at this point by maximizing the quantity and minimizing the energy. According to the theory of the brain economy (Bullmore and Sporns, 2012), the efficiency of information processing in the brain is likely optimized by trading off with energy consumption. Although biological and empirical requirements regarding efficiency and energy are independent of each other, the result indicates that they are correlated, so there may be a mechanism that controls information transfer while satisfying these two principles regarding the efficiency and the energy.

### 4.2. Network Structure and Information Transfer

Network analyses around this optimal state may explain the mechanism by which information transfer is organized in the brain. In Figure 5A, to allow topological representation, I estimated the largest component size of the network. The correlation with mutual information (Figure 5B) indicated that the efficiency of information transfer is maximized at the critical point between the fully connected network and the fragmented network state, which contains disconnected subcomponents. At this optimal state, the network maintains its fully connected structure with the minimum number of connections (Figure 6). This can be contrasted with the fragmented states, which inhibit efficient communication due to disconnections between regions. On the other hand, excess connections generate noise in the response. Therefore, in the intermediate phase at the optimal state, information transfer is cost effective, suppressing excess signals, and preserving fully connected structure.

As illustrated in Figure 6, this state can be described as the topological phase transition between the fully connected and fragmented phases. In this way, it constitutes a critical state. The distribution shape of node strength, another variable of the network, corroborates the notion that mutual information is maximized at the critical state. As shown in Figure 7A, the distribution of node strength converges in the model Equation (8), which assumes criticality and energy constraints (Takagi, 2017, 2018). This correlation shows that mutual information (Figure 7C) increases as the values converge upon the critical state. This result, along with the weighted network description (Figure 7B), also suggests that topological states are also correlated (Figures 4B,D). Both of these results indicate that the optimal state regarding the efficiency of the information transfer emerges in the critical state, suggesting that there is criticality in the brain, as has been confirmed empirically in various studies (Beggs and Plenz, 2003; Achard et al., 2006; Beggs, 2008; van den Heuvel and Sporns, 2011).

Although the state, which was specified as optimal, depends on the parameters, such as the threshold value, the criticality that supports its generality. Because the critical state was obtained without adjusting or fine-tuning multiple system parameters, it indicates that this state has the generality, which was obtained regardless of the details of the parameters. In fact, the stable results for the large samples about 1,000 individuals imply that these features around the optimal state are general ones, which emerge commonly and stably for different individuals.

This statistical features of node strength provide further information about the mechanism of the information transfer in this optimal state. The distribution of node strength exhibits a characteristic shape, as illustrated in Figure 7B. The cumulative distribution curve on the log-log plot indicates that the network contains a large number of higher strength nodes, which correspond to hubs in the functional network and comprise the core structure within networks (Hagmann et al., 2008; van den Heuvel and Sporns, 2011). Thanks to such core networks, whole networks can acquire the attributes of a small-world structure, allowing efficient communication with shortened distance between the nodes (Bassett and Bullmore, 2006) and improved robustness of information transfer.

### 4.3. Activation Patterns and Principles of Energy and Efficiency

The importance of these network states in regulating activity in the brain can be evaluated using activation patterns. According to the definition of the information transfer (Equation 2), the response signals for the random input stimuli might be determined, reflecting the network structure. For example, the response probabilities are determined by the combination of *w*_*i, j*_ ≠ 0 elements for each *i*, and then the overlapping number would be given accordingly. Then, the overlapping number was an indicator, which reflects the network structure, activation patterns, and information transfer.

In Figure 8A, I evaluated the number of overlapping activated regions between different response signals. The correlation with efficiency of information transfer and energy are shown in Figures 8B–D, which show that network structure behaves in a similar way (Figures 5B, 7C), indicating that these quantities depend strongly on the overlapping number. Increase in this quantity to the higher threshold was explained by the over-inactivated states with many 0 signals. The saturation of the activated signals, the higher density of the signals shown in Figure 3C, explains the same tendency, that is, increasing this quantity from the lower threshold. In each case, the overlapping number is increased at this state than during the intermediate states, at which activated and inactivated signals are balanced. Thus, the correlation between the mutual information entropy and the activation patterns can be explained by this quantity, the overlapping number.

As discussed above, increased overlapping may improve robustness in signal transfer and facilitate rapid response to the outer environment, with shortened communication distance between nodes. Despite these advantages, excess overlapping in the activation phase reduces the efficiency of the information transfer and causes the energy loss (Figures 8B–D). This implies that excess overlapping causes loss of efficiency and increases the energy consumption related to information transfer. Thus, these features have a trade-off relationship; that is, the robustness and the rapidity of responses are balanced with loss of efficiency and energy in information transfer.

In summary, the present results suggest that the principles of efficiency and energy consumption are important to information transfer. These principles affect multiple aspects of the functional network in the brain, and I have shown the connectivity strength (Figure 7C), activation patterns (Figures 8B–D), and topological network of such structures (Figure 5B). The same figures show the contribution of these principles to statistical quantities, in which sharp peaks indicate a strong tendency toward these quantities. Thus, these principles regarding efficiency and of information transfer are important factors in regulating the characteristic attributes of the functional network in the human brain, such as network structure and activation patterns.

## Data Availability Statement

Publicly available datasets were analyzed in this study. This data can be found here: http://umcd.humanconnectomeproject.org/.

## Author Contributions

KT designed the study, conducted the simulations and data analyses, and wrote the manuscript.

### Conflict of Interest

The author declares that the research was conducted in the absence of any commercial or financial relationships that could be construed as a potential conflict of interest.

## References

[B1] Achard S. Bullmore E. (2006). Efficiency and cost of economical brain functional networks. PLoS Comp. Biol. 3:e17. 10.1371/journal.pcbi.0030017 17274684 PMC1794324

[B2] Achard S. Salvador R. Whitcher B. Suckling J. Bullmore E. (2006). A resilient, low-frequency, small-world human brain functional network with highly connected association cortical hubs. J. Neurosci. 26, 63–72. 10.1523/JNEUROSCI.3874-05.2006 16399673 PMC6674299

[B3] Azevedo F. A. Carvalho L. R. Grinberg L. T. Farfel J. M. Ferretti R. E. Leite R. E.. (2009). Equal numbers of neuronal and nonneuronal cells make the human brain an isometrically scaled-up primate brain. J. Comp. Neurol. 513:532. 10.1002/cne.21974 19226510

[B4] Barrat A. Barthelemy M. Pastor-Satorras R. Vespignani A. (2004). The architecture of complex weighted networks. Proc. Natl. Acad. Sci. U.S.A. 101, 3747–3752. 10.1073/pnas.0400087101 15007165 PMC374315

[B5] Bassett D. Bullmore E. (2006). Small-world brain networks. Neuroscientist 12, 512–523. 10.1177/1073858406293182 17079517

[B6] Bassett D. Meyer-Lindenberg A. Achard S. Duke T. Bullmore E. (2006). Adaptive reconfiguration of fractal small-world human brain functional networks. Proc. Natl. Acad. Sci. U.S.A. 103, 19518–19523. 10.1073/pnas.0606005103 17159150 PMC1838565

[B7] Bassett D. S. Wymbs N. F. Porter M. A. Mucha P. J. Carlson J. M. Grafton S. T. (2018). Dynamic reconfiguration of human brain networks during learning. Proc. Natl. Acad. Sci. U.S.A. 118, 7641–7646. 10.1073/pnas.1018985108 PMC3088578 21502525

[B8] Beggs J. (2008). The criticality hypothesis: how local cortical networks might optimize information processing. Philos. Trans. A Math. Phys. Eng. Sci. 366, 329–343. 10.1098/rsta.2007.2092 17673410

[B9] Beggs J. Plenz D. (2003). Neuronal avalanches in neocortical circuits. J. Neurosci. 23, 11167–11177. 10.1523/JNEUROSCI.23-35-11167.2003 14657176 PMC6741045

[B10] Biswal B. Mennes M. Zuo X.-N. Gohel S. Kelly C. Smith S. M.. (2010). Toward discovery science of human brain function. Proc. Natl. Acad. Sci. U.S.A. 107, 4734–4739. 10.1073/pnas.0911855107 20176931 PMC2842060

[B11] Bressler S. Menon V. (2010). Large-scale brain networks in cognition: emerging methods and principles. Trends Cogn. Sci. 14, 277–290. 10.1016/j.tics.2010.04.004 20493761

[B12] Brown J. Rudie J. Bandrowski A. Van Horn J. Bookheimer S. (2012). The UCLA multimodal connectivity database: a web-based platform for brain connectivity matrix sharing and analysis. Front. Neuroinform. 6:28. 10.3389/fninf.2012.00028 23226127 PMC3508475

[B13] Bullmore E. Sporns O. (2012). The economy of brain network organization. Nat. Rev. Neurosci. 13, 336–349. 10.1038/nrn3214 22498897

[B14] Calhoun V. Liu J. Adali T. (2009). A review of group ica for fMRI data and ica for joint inference of imaging, genetic, and ERP data. Neuroimage 45:S163. 10.1016/j.neuroimage.2008.10.057 19059344 PMC2651152

[B15] Chialvo D. (2010). Emergent complex neural dynamics. Nat. Phys. 6, 744–750. 10.1038/nphys1803

[B16] Clark A. (2013). Whatever next? Predictive brains, situated agents, and the future of cognitive science. Behav. Brain Sci. 36:181–204. 10.1017/S0140525X12000477 23663408

[B17] Clauset A. Shalizi C. Newman M. (2009). Power-law distributions in empirical data. SIAM Rev. 51, 661–703. 10.1137/070710111

[B18] Eguiluz V. Chialvo D. Cecchi G. Baliki M. Apkarian A. (2005). Scale-free brain functional networks. Phys. Rev. Lett. 94:018102. 10.1103/PhysRevLett.94.018102 15698136

[B19] Fenno L. Yizhar O. Deisseroth K. (2011). The development and application of optogenetics. Annu. Rev. Neurosci. 34:389. 10.1146/annurev-neuro-061010-113817 21692661 PMC6699620

[B20] Finn E. Shen X. Scheinost D. Rosenberg M. Huang J. Chun M. M. (2015). Functional connectome fingerprinting: identifying individuals based on patterns of brain connectivity. Nat. Neurosci. 18:1664 10.1038/nn.4135 26457551 PMC5008686

[B21] Fox M. Raichle M. (2007). Spontaneous fluctuations in brain activity observed with functional magnetic resonance imaging. Nat. Rev. Neurosci. 8, 700–711. 10.1038/nrn2201 17704812

[B22] Fox M. Snyder A. Vincent J. Corbetta M. Van Essen D. Raichle M. (2005). The human brain is intrinsically organized into dynamic, anticorrelated functional networks. Proc. Natl. Acad. Sci. U.S.A. 102, 9673–9678. 10.1073/pnas.0504136102 15976020 PMC1157105

[B23] Friston K. (2010). The free-energy principle: a unified brain theory? Nat. Rev. Neurosci. 11, 127–138. 10.1038/nrn2787 20068583

[B24] Ghazanfar A. Schroeder C. (2006). Is neocortex essentially multisensory? Trends Cogn. Sci. 10, 278–285. 10.1016/j.tics.2006.04.008 16713325

[B25] Greicius M. Supekar K. Menon V. Dougherty R. (2009). Restingstate functional connectivity reflects structural connectivity in the default mode network. Cereb. Cortex 19, 72–78. 10.1093/cercor/bhn059 18403396 PMC2605172

[B26] Hagmann P. Cammoun L. Gigandet X. Meuli R. Honey C. Wedeen V. J.. (2008). Mapping the structural core of human cerebral cortex. PLoS Biol. 6:e159. 10.1371/journal.pbio.0060159 18597554 PMC2443193

[B27] Haxby J. Gobbini M. I. Furey M. L. Ishai A. Schouten J. L. Pietrini P. (2001). Distributed and overlapping representations of faces and objects in ventral temporal cortex. Science 293, 2425–2430. 10.1126/science.1063736 11577229

[B28] Hilgetag C. Grant S. (2000). Uniformity, specificity and variability of corticocortical connectivity. Philos. Trans. R. Soc. Lond. B Biol. Sci. 355, 7–20. 10.1098/rstb.2000.0546 10703041 PMC1692717

[B29] Hinton G. E. Salakhutdinov R. R. (2006). Reducing the dimensionality of data with neural networks. Science 313, 504–507. 10.1126/science.1127647 16873662

[B30] Hopfield J. J. (1984). Neurons with graded response have collective computational properties like those of two-state neurons. Proc. Natl. Acad. Sci. U.S.A. 81, 3088–3092. 10.1073/pnas.81.10.3088 6587342 PMC345226

[B31] Kanwisher N. McDermott J. Chun M. (1997). The fusiform face area: a module in human extrastriate cortex specialized for face perception. J. Neurosci. 17, 4302–4311. 10.1523/JNEUROSCI.17-11-04302.1997 9151747 PMC6573547

[B32] Kitzbichler M. G. Smith M. Christensen S. Bullmore E. (2009). Broadband criticality of human brain network synchronization. PLoS Comput. Biol. 5:e1000314. 10.1371/journal.pcbi.1000314 19300473 PMC2647739

[B33] Klaus A. Yu S. Plenz D. (2011). Statistical analyses support power law distributions found in neuronal avalanches. PLoS ONE 6:e19779. 10.1371/journal.pone.0019779 21720544 PMC3102672

[B34] Kumaran D. Hassabis D. McClelland J. (2016). What learning systems do intelligent agents need? Complementary learning systems theory updated. Trends Cogn. Sci. 20, 512–534. 10.1016/j.tics.2016.05.004 27315762

[B35] Lee J. H. Durand R. Gradinaru V. Zhang F. Goshen I. Kim D.-S.. (2010). Global and local fmri signals driven by neurons defined optogenetically by type and wiring. Nature 465:788. 10.1038/nature09108 20473285 PMC3177305

[B36] Linsker R. (1990). Perceptual neural organisation: some approaches based on network models and information theory. Annu. Rev. Neurosci. 13, 257–281. 10.1146/annurev.ne.13.030190.001353 2183677

[B37] Meunier D. Lambiotte R. Bullmore E. (2010). Modular and hierarchically modular organization of brain networks. Front. Neurosci. 4:200. 10.3389/fnins.2010.00200 21151783 PMC3000003

[B38] Mnih V. Kavukcuoglu K. Silver D. Rusu A. Veness J. Bellemare M.. (2015). Human-level control through deep reinforcement learning. Nature 518, 529–533. 10.1038/nature14236 25719670

[B39] Niven J. Laughlin S. (2008). Energy limitation as a selective pressure on the evolution of sensory systems. J. Exp. Biol. 211, 1792–1804. 10.1242/jeb.017574 18490395

[B40] Park H. Friston K. (2013). Structural and functional brain networks: from connections to cognition. Science 342:1238411. 10.1126/science.1238411 24179229

[B41] Rubinov M. Sporns O. (2011). Weight-conserving characterization of complex functional brain networks. Neuroimage 56, 2068–2079. 10.1016/j.neuroimage.2011.03.069 21459148

[B42] Shmuel A. Augath M. Oeltermann A. Logothetis N. (2006). Negative functional mri response correlates with decreases in neuronal activity in monkey visual area v1. Nat. Neurosci. 9, 569–577. 10.1038/nn1675 16547508

[B43] Smith S. (2012). The future of fMRI connectivity. Neuroimage 62:1257. 10.1016/j.neuroimage.2012.01.022 22248579

[B44] Smith S. Miller K. Salimi-Khorshidi G. Webster M. (2011). Network modelling methods for fMRI. Neuroimage 54:875. 10.1016/j.neuroimage.2010.08.063 20817103

[B45] Song B. Ma N. Liu G. Zhang H. Yu L. Liu L.. (2019). Maximal flexibility in dynamic functional connectivity with critical dynamics revealed by fMRI data analysis and brain network modelling. J. Neural Eng. 16:056002. 10.1088/1741-2552/ab20bc 31071699

[B46] Sporns O. (2002). Network analysis, complexity, and brain function. Complexity 8, 56–60. 10.1002/cplx.10047

[B47] Sporns O. (2013). Network attributes for segregation and integration in the human brain. Curr. Opin. Neurobiol. 23, 162–171. 10.1016/j.conb.2012.11.015 23294553

[B48] Sporns O. Tononi G. Kotter R. (2005). The human connectome: a structural description of the human brain. PLoS Comput. Biol. 1:e42. 10.1371/journal.pcbi.0010042 16201007 PMC1239902

[B49] Tagliazucchi E. Balenzuela P. Fraiman D. Chialvo D. (2012). Criticality in large-scale brain fMRI dynamics unveiled by a novel point process analysis. Front. Physiol. 3:15. 10.3389/fphys.2012.00015 22347863 PMC3274757

[B50] Tagliazucchi E. Chialvo D. Siniatchkin M. Amico E. Brichant J.-F. Bonhomme V.. (2016). Large-scale signatures of unconsciousness are consistent with a departure from critical dynamics. J. R. Soc. Interface 13:20151027. 10.1098/rsif.2015.1027 26819336 PMC4759808

[B51] Takagi K. (2017). A distribution model of functional connectome based on criticality and energy constraints. PLoS ONE 12:e0177446. 10.1371/journal.pone.0177446 28545048 PMC5435242

[B52] Takagi K. (2018). Information-based principle induces small-world topology and self-organized criticality in a large scale brain network. Front. Comp. Neurosci. 12:65. 10.3389/fncom.2018.00065 30131688 PMC6090464

[B53] Tomasi D. Wang G.-J. Volkow N. (2013). Energetic cost of brain functional connectivity. Proc. Natl. Acad. Sci. U.S.A. 110, 13642–13647. 10.1073/pnas.1303346110 23898179 PMC3746878

[B54] Tononi G. Sporns O. Edelman G. (1994). A measure for brain complexity: relating functional segregation and integration in the nervous system. Proc. Natl. Acad. Sci. U.S.A. 91:5033. 10.1073/pnas.91.11.5033 8197179 PMC43925

[B55] van den Heuvel M. Sporns O. (2011). Rich-club organization of the human connectome. J. Neurosci. 31, 15775–15786. 10.1523/JNEUROSCI.3539-11.2011 22049421 PMC6623027

[B56] van den Heuvel M. Stam C. Boersma M. HulshoffPol H. (2008). Small world and scale-free organization of voxel based resting-state functional connectivity in the human brain. Neuroimage 43, 528–539. 10.1016/j.neuroimage.2008.08.010 18786642

[B57] Van Dijk K. Hedden T. Venkataraman A. Evans K. Lazar S. (2010). Intrinsic functional connectivity as a tool for human connectomics: theory, properties, and optimization. J. Neurophysiol. 103, 297–321. 10.1152/jn.00783.2009 19889849 PMC2807224

[B58] Wang R. Tsuda I. Zhang Z. (2015). A new work mechanism on neuronal activity. Int. J. Neural Syst. 25:1450037. 10.1142/S0129065714500373 25640576

[B59] Wang R. Zhang Z. Chen G. (2008). Energy function and energy evolution on neural population. IEEE Trans. Neural Netw. 19, 535–538. 10.1109/TNN.2007.914177 18334373

[B60] Wang Y. Xu X. Wang R. (2018). An energy model of place cell network in three dimensional space. Front. Neurosci. 12:264. 10.3389/fnins.2018.00264 29922119 PMC5996932

[B61] Wang Z. Wang R. (2014). Energy distribution property and energy coding of a structural neural network. Front. Comp. Neurosci. 8:14. 10.3389/fncom.2014.00014 24600382 PMC3930871

[B62] Watts D. Strogatz S. (1998). Collective dynamics of ‘small-world' networks. Nature 393, 440–442. 10.1038/30918 9623998

[B63] Whitacre J. M. (2010). Degeneracy: a link between evolvability, robustness and complexity in biological systems. Theor. Biol. Med. Model. 7:6. 10.1186/1742-4682-7-6 20167097 PMC2830971

[B64] Yu L. Yu Y. (2017). Energy–efficient neural information processing in individual neurons and neuronal networks. J. Neurosci. Res. 95, 2253–2266. 10.1002/jnr.24131 28833444

[B65] Zuo X.-N. Ehmke R. Mennes M. Imperati D. Castellanos F. Sporns O.. (2012). Network centrality in the human functional connectome. Cereb. Cortex 22, 1862–1875. 10.1093/cercor/bhr269 21968567

